# A Female With Synchronous Multiple Primary Malignant Tumors in the Esophagogastric Junction, Duodenum and Pancreas: Case Report and Review of the Literature

**DOI:** 10.3389/fonc.2022.890587

**Published:** 2022-05-30

**Authors:** Yongxing Du, Yunjie Duan, Lipeng Zhang, Zongting Gu, Xiaohao Zheng, Zongze Li, Chengfeng Wang

**Affiliations:** ^1^ State Key Laboratory of Molecular Oncology and Department of Pancreatic and Gastric Surgery, National Cancer Center/National Clinical Research Center for Cancer/Cancer Hospital, Chinese Academy of Medical Sciences and Peking Union Medical College, Beijing, China; ^2^ Department of Burns and Plastic Surgery, The Fourth Medical Center of PLA General Hospital, Beijing, China

**Keywords:** pancreas, multiple primary tumors, comprehensive treatment, literature review, prognostic analysis

## Abstract

The incidence of multiple primary carcinomas (MPCs), which are defined as two or more malignancies detected in an individual person, is gradually increasing around the world. According to the timing of diagnosis for each constituent tumor, MPCs are classified into 2 categories: synchronous MPCs if constituent tumors emerge simultaneously or within 6 months or metachronous MPCs otherwise. In this report, we describe our recent observation and treatment of a female patient with synchronous primary esophagogastric junction adenocarcinoma, duodenal adenocarcinoma and pancreatic ductal adenocarcinoma (PDAC). To the best of our knowledge, this combination has not yet been reported in the literature. A crucial aspect is the decision regarding which tumor to treat initially and how to schedule further treatments according to individual tumor hazards. Our multidisciplinary team devised an individualized treatment regimen for this patient. The patient ultimately achieved an overall survival time of 18 months, which was much longer than the median survival time (6~11 months) of patients with locally advanced pancreatic cancer. Moreover, treating this rare combination raised a series of diagnostic, etiological and therapeutic questions, motivating us to carry out a critical review of the literature. In summary, an individualized treatment strategy with input from a dedicated multidisciplinary team and consideration of all options at different points along the disease trajectory is essential to optimize outcomes for patients with MPC.

## Introduction

MPC, first reported by Billroth in 1879, refers to the simultaneous or subsequent occurrence of two or more cancers unrelated to each other in one patient, which may occur in different parts of the same organ or the same system or in different organs or systems (1). In recent research, MPC was defined by Moertel as two or more malignant tumors occurring within 6 months (2). The burden of MPCs is rising as the aging population has increased over the last decades (2). Several studies have retrospectively investigated the incidence of MPCs. For example, the study of Alexia et al. showed that the incidence of MPCs in a cancer population varies between 2.4% and 8% within 20 years of follow-up (3). Moreover, another study from data of European cancer registries reported an overall incidence of multiple primary cancers of 6.3% (range, 0.4–12.9%) (4).

With the advancement of medical technology and improvements in comprehensive clinical diagnosis and treatment, previously difficult-to-find tumor lesions can now be detected easily, thereby increasing the chance of detecting multiple primary cancers. Nevertheless, owing to its low prevalence, many clinicians are not sufficiently aware of this disease and lack therapeutic experience. To date, the pathogenesis of this rare disease remains to be elucidated. MPC is clinically often confused with the recurrence or metastasis of malignant tumors which might greatly change the formula of patients. In view of above situation, we decided to report our recent observation and treatment of a female patient with synchronous primary esophagogastric junction adenocarcinoma, duodenal adenocarcinoma and PDAC. To the best of our knowledge, this combination has not yet been reported in the literature.

## Case Report

On January 23, 2019, a 77-year-old female patient was admitted to the Cancer Hospital, Chinese Academy of Medical Sciences, because of mild abdominal pain and intermittent fever (with a maximum temperature of 39°C) for more than 3 months, and a duodenal mass was detected 2 weeks prior. Moreover, she presented with transient jaundice and skin itching that lasted for approximately 5 days. She did not complain of any other discomfort and did not report any relevant family history, and she had not been treated at a nearby clinic or hospital. Because of the noninvasive, radiation-free, and convenient characteristics of abdominal ultrasound, the patient had a habit of regular medical examination with abdominal ultrasound every year. The patient’s duodenal mass was diagnosed by ultrasound during her annual physical examination two weeks prior, then she came to our hospital for further tests and treatment.

**Figure 1:**
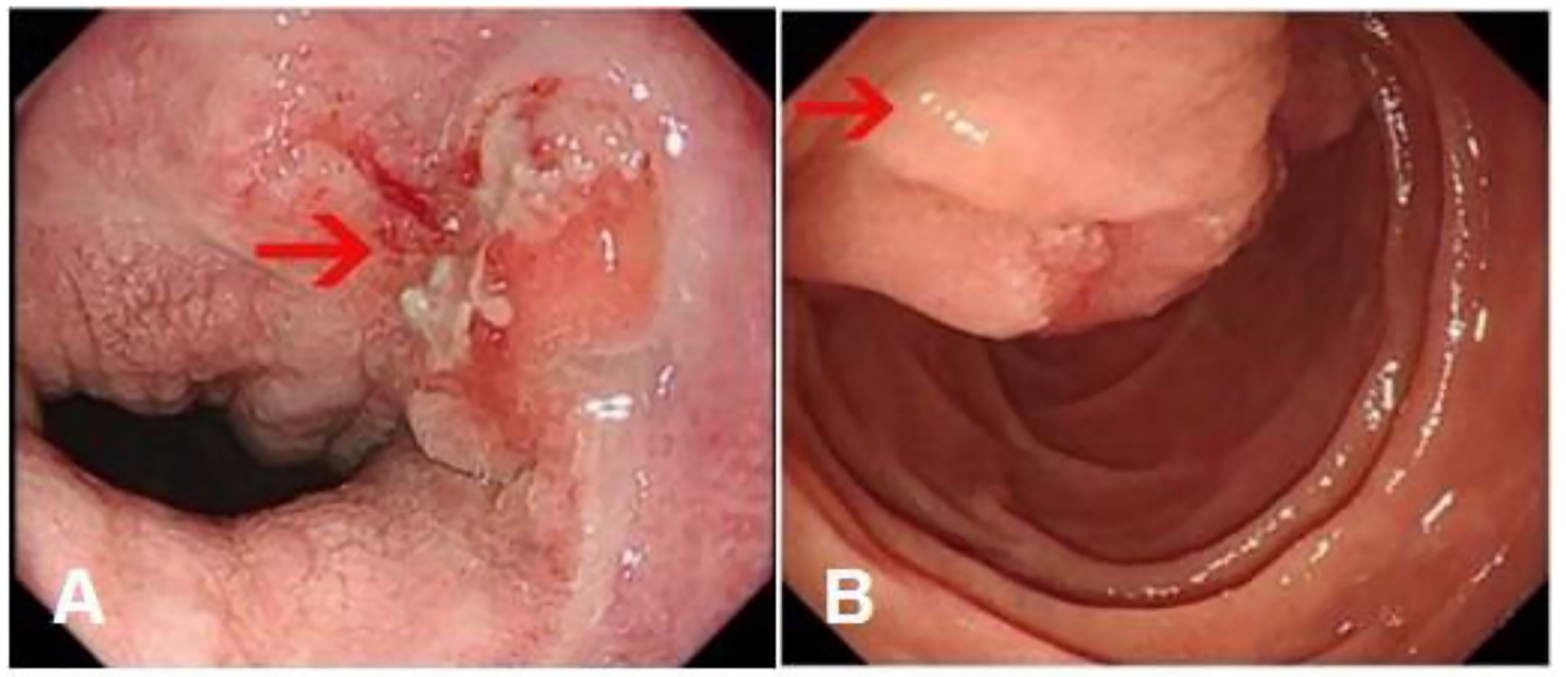
Gastroscopy results. **(A)** Superficial ulceration at the esophagogastric junction with irregular protrusion. (Arrow: the location of the tumor) **(B)** A polypoidal protuberant mass in the duodenal papilla protruding into the lumen. (Arrow: the location of the tumor).

**Figure 2:**
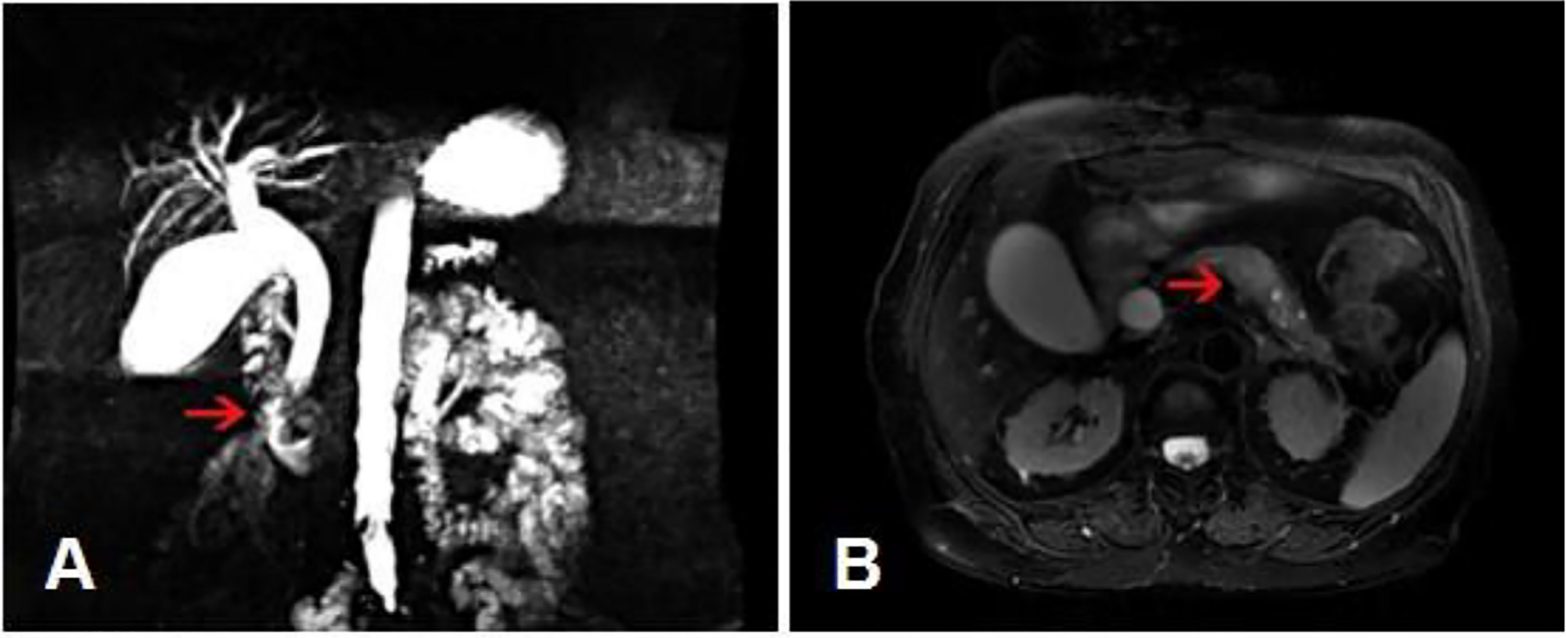
MRCP results. **(A)** A soft tissue nodule at the end of the common bile duct with low biliary tract obstruction. (Arrow: the location of the tumor). **(B)** A poorly defined somatic nodule at the body of the pancreas. (Arrow: the location of the tumor).

**Figure 3:**
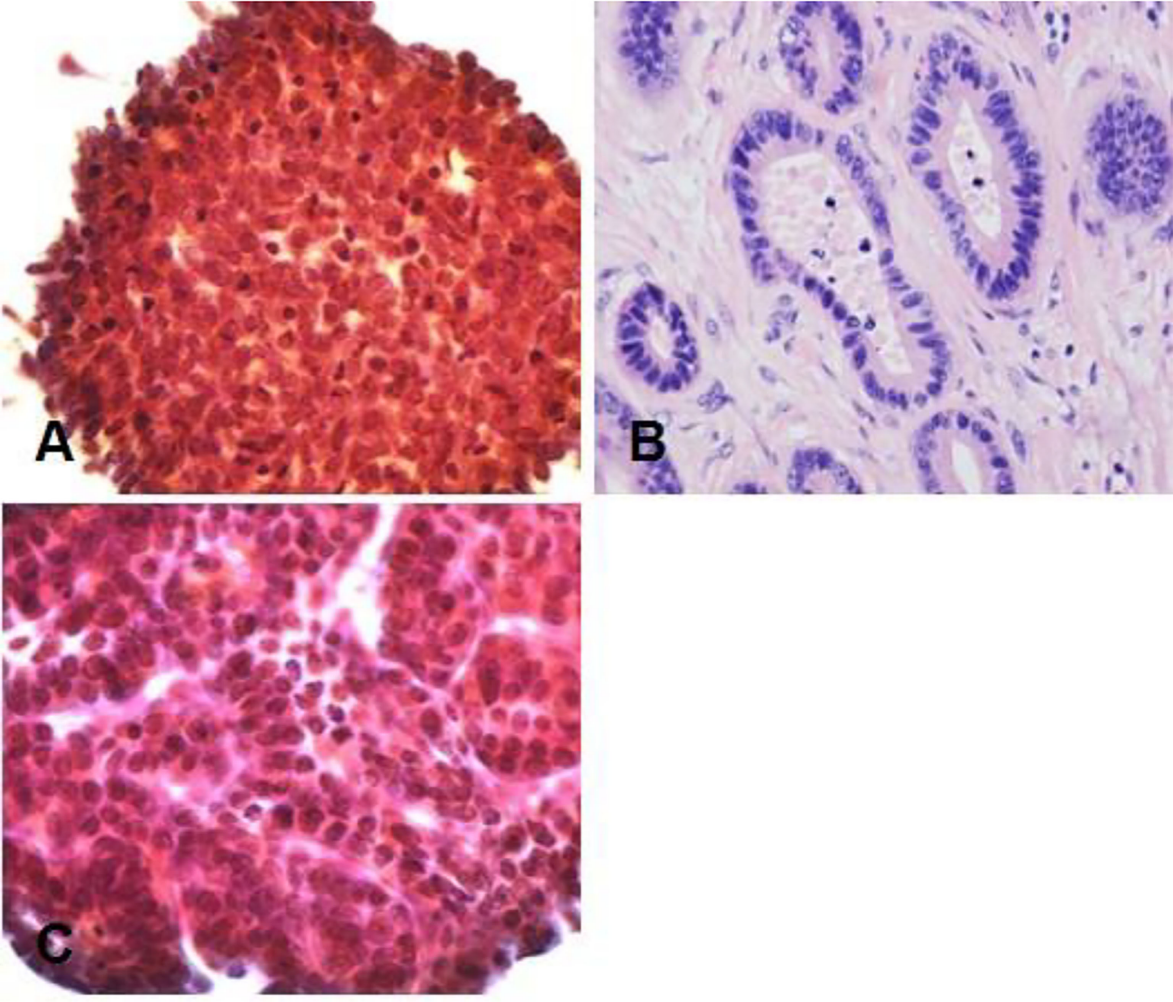
Pathological results. **(A)** Duodenal adenocarcinoma. **(B)** Moderately differentiated adenocarcinoma (Lauren classification: intestinal type) at the esophagogastric junction. **(C)** Pancreatic adenocarcinoma at the pancreatic body. Hematoxylin–eosin (H&E) staining of biopsy samples (40×) magnification.

Gastroscopy revealed superficial ulceration at the esophagogastric junction with irregular protrusion and a polypoidal protuberant mass in the duodenal papilla protruding into the lumen (**Figure 1**). Pancreatic magnetic resonance cholangiopancreatography (MRCP) reported soft tissue nodules at the end of the common bile duct approximately 2.6x1.7 cm in size that were prone to malignancy, with low biliary tract obstruction (**Figure 2A**). Another pancreatic somatic nodule was found in the body of the pancreas that was approximately 2.0x2.3 cm in size and poorly defined, so the possibility of malignancy could not be excluded (**Figure 2B**). The pathological results of endoscopic biopsy revealed adenocarcinoma in both lesions of the esophagogastric junction and duodenal papilla (**Figure 3A**). Hematologic examination revealed the following: ALT(88 U/L), ALP(280 u/L), DBIL(5.7 µmol/L), CA199(551 U/ml), and CA242(107.190 U/ml).

The patient was 159 cm tall and weighed 70.0 kg, and her body mass index (BMI) was 27.7. She had a past history of hypertension, type-2 diabetes mellitus, and osteoarthritis. More than 20 years ago, she underwent thyroid surgery, and the pathology was benign. After the operation, the patient was treated with oral thyroid hormone drugs.

## Diagnostic Investigation and Treatment

Although the definition and criteria for the diagnosis of MPC have changed many times over the years, it is typically based on the criteria described by Warren and Gates (5): (a) each tumor must have a clear pathological diagnosis; (b) each tumor must have an independent initial site of disease; and (c) the lesions must be nonmetastatic tumors. We initially determined that this case fulfilled the diagnostic criteria for MPC by the above medical examinations, and then the case was discussed by a multidisciplinary team before treatment was initiated. Since the patient’s family refused pancreatoduodenectomy due to the high risk associated with the operation, our multidisciplinary team recommended that the tumors of the gastroesophageal junction be treated by endoscopic submucosal dissection because of the relatively early disease stage. For the pancreatic lesions and duodenal mass, the team recommended abdominal section exploration combined with intraoperative puncture biopsy of the pancreatic tumor, intraoperative radiotherapy, and gallbladder jejunostomy and gastrojejunostomy due to the high risk of radical surgery and to respect the wishes of the patient and her family.

The patient underwent two operations in our hospital. The first operation was endoscopic submucosal dissection (ESD) performed under general anesthesia on February 19, 2019. The final pathological result showed moderately differentiated adenocarcinoma (Lauren classification: intestinal type) (**Figure 3B**). It had invaded the submucosa at a depth of 1550 microns (1650 microns) and was staged as pT1b. Immunohistochemical results showed the following: AFP (-), CD10 (intramucosal +, submucosal region +, glandular surface +, partial intramucosal -), cdx-2 (2+), c-met (2+), EGFR(2+), GPC3(-), HER2(2+), MLH1(+), MSH2(+), MSH6(+), MUC2(surface 1+, deep -), MUC5AC(1+), MUC6(1+), P53(90%+, missense mutated expression), SALL4(-), CD31, D2-40 (vascular staining), and Desmin (muscularis mucosa staining). Special staining results were observed for elastic fiber staining (showing veins). Gene detection did not show mutations in KRAS gene exons 2, 3 and 4, NRAS gene exons 2, 3 and 4, PIK3CA gene exons 9 and 20, or BRAF gene exons 11 and 15. On March 4, 2019, abdominal exploration was performed under general anesthesia. Intraoperative exploration revealed an unresectable tumor (the tumor was closely adhered to the mesenteric vasculature, and their separation was not possible) approximately 3x4 cm in size in the pancreatic body. The intraoperative pancreatic biopsy result was suggestive of adenocarcinoma (**Figure 3C**). Therefore, intraoperative radiotherapy with a dosage of 1500 cGy was delivered to the pancreatic mass. Gallbladder jejunostomy and gastrojejunostomy were also performed to relieve the obstruction of the biliary and gastrointestinal tracts. The final results of biopsy immunohistochemistry showed the following: braf-v600e (-), c-met (2+), HER2(1+), MLH1(+), MSH2(+), MSH6(+), PMS2(+), AE1/AE3(3+), CK18(3+), ChrA(-), Syno(-), and Ki-67 (30%+). In combination with the immunohistochemistry results, it was suggested that there was little infiltration of poorly differentiated adenocarcinoma into the fibrous adipose tissue with mucous secretion.

**Figure 4:**
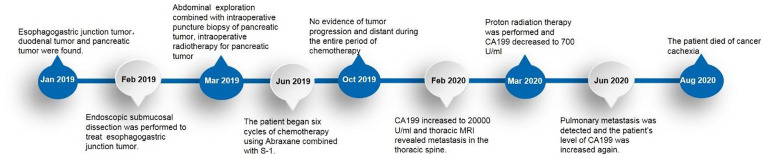
Timeline of MPC progression in our female patient.

The patient recovered well, and she was discharged on the 14th postoperative day after the second operation. The first month after surgery, the patient had resumed her preoperative diet, her abdominal pain significantly improved, and no fever was reported. From June 10 to October 21, 2019, the patient underwent six cycles of chemotherapy (Abraxane combined with S-1) at the internal medicine department of our hospital. S-1, also known as Tegafur, Gimeracil and Oteracil Porassium Capsules, is a fluorouracil derivative oral anticancer agent that includes tegafur and the following two classes of modulators: gimeracil and oteracil. This medicine is mainly used for the treatment of unresectable locally advanced or metastatic gastric cancer and pancreatic cancer, etc. (6). After six courses of chemotherapy, her tumor markers were still not normalized (CEA, 15.25 ng/ml; CA199, 580.6 U/ml; CA242, >200 U/ml). However, no evidence of tumor progression or distant metastasis was found on multiple follow-up chest and abdominal CT and MRI scans during the entire period of chemotherapy. Four months later, the patient’s level of CA199 increased to 20000 U/ml, and thoracic MRI revealed metastasis in the thoracic spine. Then, she received for further treatment in the form of proton radiation therapy at another hospital in February 2020. Radiotherapy achieved some encouraging treatment outcomes, and the patient’s CA199 level decreased to 700 U/ml. Unfortunately, pulmonary metastasis was detected in June 2020, and the patient’s level of CA199 had increased again. The patient ultimately died of cancer cachexia on August 27, 2020 (**Figure 4**).

## Discussion and Literature Review

Liu reported that MPC most commonly occurs in the respiratory system and gastrointestinal tract, while intracranial origin is rare (1). Utada stated that esophageal cancer and oral/pharyngeal cancer are the most common types of MPC (5). Other studies have reported that the incidence of multiple primary cancers in the digestive system is 2% ~ 17%, and that of multiple primary cancer patients with pancreatic cancer is 5.2% ~ 23% (7–12). To gain further insights into the occurrence of pancreatic cancer with multiple primary cancers, we carried out a critical review of the literature. A summary of the previous studies describing MPC is shown in **Table 1**.

**Table 1:** Summary of the previous studies describing multiple primary malignant neoplasms with pancreatic cancer.

Year	Author	1st Tumor	2nd Tumor	3rd Tumor	4th Tumor	Synchronous or Metachronous primary cancers	Case report or Retrospective cohort study	Number of patients	Outcomes	Genomic findings
1965	M Sasaki	Pancreatic carcinoma	Hepatocellular carcinoma			Synchronous	Case report	1	–	–
1966	P N Bodnar	Lung carcinoma	Pancreatic carcinoma			Synchronous	Case report	1	–	–
1969	E Sasaki	Pancreatic carcinoma	Renal carcinoma			Metachronous	Case report	1	The patient died of cachexia.	–
1972	P Langeron	Cervical carcinoma	Pancreatic carcinoma			Synchronous	Case report	1	–	–
1983	Y Kawaura	Pancreatic carcinoma	Appendiceal tumor			Synchronous	Case report	1	The patient died of cachexia.	–
1985	N Hori	Bladder carcinoma	Prostatic carcinoma	Pancreatic carcinoma		Synchronous	Case report	4	–	–
1986	M E O’Brien	Pancreatic carcinoma	Breast carcinoma			Synchronous	Case report	2	Patient 1 died of cardiac arrest. Patient 2 died of cachexia.	–
1987	Y Niv	Pancreatic carcinoma	Duodenal carcinoma			Synchronous	Case report	1	The patient died of liver failure.	–
1988	Niwa K	Pancreatic carcinoma	Ovarian carcinoma			Synchronous	Case report	1	–	–
1989	Yoshii K	Pancreatic carcinoma	Biliary tract carcinoma			Synchronous	Case report	1	The patient died of respiratory failure.	–
1990	A G Montag	Pancreatic adenocarcinoma	Pancreatic sarcoma			Synchronous	Case report	2	Patient 1 died of hypotensive shock. Patient 2 died of metastatic tumor.	–
1990	L C Childs	Biliary tract carcinoma	Pancreatic carcinoma			Synchronous	Case report	1	The patient died of cachexia.	–
1992	N Ueda	Biliary tract carcinoma	Pancreatic carcinoma			Synchronous	Case report	1	11 months without evidence of recurrence.	–
1994	Nishihara K	Pancreatic carcinoma	carcinoma of the papilla of Vater	Biliary tract carcinoma		Synchronous	Case report	1	The patient was still alive and well 4 years and 2 months after surgery.	The overexpression of p53 in the three tumors of the present case was found. / The DNA of the tumors in the present case were all aneuploid.
1994	P K Karak	Colonic carcinoma	Pancreatic carcinoma			Synchronous	Case report	1	The patient died of metastatic tumor.	–
2000	Eriguchi N	Pancreatic carcinoma	Gastric carcinoma			Synchronous	Case report	3	The patient died of cachexia.	–
Table 1. Continued										
Year	Author	1st Tumor	2nd Tumor	3rd Tumor	4th Tumor	Synchronous or Metachronous primary cancers	Case report or Retrospective cohort study	Number of patients	Outcomes	Genomic findings
2000	Eriguchi N	Gastric carcinoma	Pancreatic carcinoma			Synchronous	Case report	2	The patient died of cachexia.	–
2000	Eriguchi N	Lung carcinoma	Pancreatic carcinoma			Synchronous	Case report	2	The patient died of cachexia.	–
2000	Eriguchi N	Colonic carcinoma	Pancreatic carcinoma			Synchronous	Case report	2	The patient died of cachexia.	–
2001	Joo YE	Pancreatic carcinoma	Colonic carcinoma			Synchronous	Case report	1	–	–
2003	Sato K	Gallbladder carcinoma	Biliary tract carcinoma	Pancreatic carcinoma		Synchronous	Case report	1	The patient died of cachexia.	The presence of p53-positive nuclei was recognized in the pancreatic cancer specimen.
2004	Brinster DR	Colonic carcinoma	Pancreatic carcinoma			Synchronous	Case report	1	–	A germline mutation of the STK11/LKB1 tumor suppressor gene was recognized.
2004	Olgyai G	Renal carcinoma	Pancreatic carcinoma			Synchronous	Case report	1	–	–
2006	I M’sakni	Pancreatic carcinoma	Gastrointestinal stromal tumor			Synchronous	Case report	2	The patients died of cachexia.	–
2008	Aurello P	Pancreatic adenocarcinoma	Pancreatic sarcoma			Synchronous	Case report	1	–	–
2010	Muroni M	Gastric carcinoma	Pancreatic carcinoma			Synchronous	Case report	1	The patients died of metastatic tumor.	–
2008	Aurello P	Pancreatic adenocarcinoma	Pancreatic sarcoma			Synchronous	Case report	1	–	–
2010	Ozsoy O	Prostatic carcinoma	Pancreatic carcinoma			Synchronous	Retrospective cohort study	419	In patients with prostate cancer, abdominopelvic CT staging detects incidental second primary cancers with a greater frequency than that expected.	–
2011	Kenichiro Araki	Pancreatic carcinoma	Renal carcinoma			Synchronous	Case report	1	52 months without evidence of recurrence after the surgery.	–
2011	Maurea S	Pancreatic neuroendocrine carcinoma	Biliary tract carcinoma			Synchronous	Case report	1	–	–
2011	Power DG	Pancreatic adenocarcinoma	Pancreatic neuroendocrine carcinoma			Synchronous	Case report	2	Patient 1 is unknown. /Patient 2 succumbed to progressive disease 20 months after an initial diagnosis.	–
2011	Dasanu CA	Pancreatic carcinoma	Gastrointestinal stromal tumor			Synchronous	Case report	1	14 months without evidence of recurrence after the surgery.	–
2011	Gyorki DE	Esophageal adenocarcinom	Gastrointestinal stromal tumor			Synchronous	Case report	1	–	–
2013	Kim JS	Thyroid Carcinoma	Breast carcinoma	Pancreatic carcinoma	Gastric carcinoma	Metachronous	Case report	1	The patients died of cachexia.	–
2013	Kourie HR	Pancreatic carcinoma	Gastric carcinoma			Metachronous	Case report	2	–	The tumors of this syndrome demonstrate loss of protein expression of mismatch repair genes and are associated with microsatellite instability (MSI).
2014	Li Destri G	Colonic carcinoma	Pancreatic carcinoma			Synchronous	Case report	1	The patients died of metastatic tumor.	–
2015	Ghothim M	Pancreatic carcinoma	Gastric carcinoma			Synchronous	Case report	1	The patient survived for 12 months.	–
2015	Ghothim M	Pancreatic carcinoma	Renal carcinoma			Synchronous	Case report	1	The patient survived for 19 months.	–
2016	Bansal A	Pancreatic carcinoma	Biliary tract carcinoma			Synchronous	Case report	1	–	–
2018	Vijayaraj P	Gallbladder carcinoma	Pancreatic carcinoma			Synchronous	Case report	1	16 months without evidence of significant metastasis-related symptoms.	–
2019	Couch LL	Colonic carcinoma	Pancreatic carcinoma			Synchronous	Case report	1	–	–
2020	Wang jun Zhang	Hepatocellular carcinoma	Pancreatic carcinoma			Synchronous	Case report	1	2 years without evidence of recurrence after the surgery.	–

### Pancreatic Carcinoma and Hepatocellular Carcinoma

We retrieved two articles focusing on primary pancreatic carcinoma combined with hepatocellular carcinoma, and all cases were synchronous MPCs (13, 14) Sasaki and Zhang performed both hepatectomy and pancreaticoduodenectomy during the course of therapy. In the case report of Zhang, the patient received chemotherapy with gemcitabine combined with tegafur gimeracil oteracil potassium and anti-hepatitis C virus (HCV) therapy after the operation. Zhang investigated the possibility that HCV infection could increase the incidence of pancreatic cancers, but the biological mechanism underlying HCV-induced pancreatic cancers has not been fully elucidated. Finally, he highlighted the critical role of PET-CT as a tumor-related systemic examination method in the diagnosis and treatment of MPC.

### Pancreatic Carcinoma and Biliary Carcinoma

Eight articles on synchronous pancreatic and biliary carcinomas were retrieved (15–22). Nishihara performed total pancreatectomy and extended cholecystectomy with regional lymph node dissection. The follow-up results showed that the patients survived for 3 years, and the recurrence-free survival time was 11 months without any adjuvant therapy after surgery. Sato adopted pancreaticoduodenectomy to treat MPC. Interestingly, the overexpression of p53 in the tumors observed in the present case was also reported by Nishihara, and he speculated that the oncogenic mechanisms of multiple synchronous cancers were caused by mutation of the p53 gene and abnormal DNA reparative mechanisms.

### Pancreatic Carcinoma and Gastric Carcinoma

We retrieved seven articles on synchronous pancreatic and gastric carcinomas (23–29). The FOLFIRINOX chemotherapy regimen was adopted to treat synchronous pancreatic and gastric cancers in the study by Kouria. He planned to re-evaluate the surgical feasibility after two months of treatment. Meanwhile, Kourie emphasized that multiple tumors demonstrated loss of protein expression of mismatch repair genes and were associated with microsatellite instability (MSI). Therefore, MSI testing may be reasonable for patients with synchronous pancreatic and gastric cancers. For patients with synchronous pancreatic carcinoma and gastrointestinal stromal tumor (GIST), pancreatectomy combined with postoperative concurrent chemoradiotherapy was performed in the study by Dasanu, and the patient was free of recurrence for 18 months following comprehensive treatment. Moreover, Dasanu suggested that screening for second cancers was warranted in patients with gastrointestinal stromal tumor (GIST) and early diagnosis of these cancerous lesions. A thorough search for second cancers, along with a multidisciplinary treatment approach, could further prolong patient survival and improve quality of life.

### Pancreatic Carcinoma and Breast Carcinoma

Two articles on synchronous pancreatic and breast carcinomas were retrieved (27, 30). Among the cases previously reported, O’Brien performed modified radical mastectomy and palliative bypass procedures to treat breast carcinoma and pancreatic carcinoma, respectively. In his case report, he implied that high dietary intake of unsaturated fats was a carcinogenic factor for both carcinomas. If there is an association between breast and pancreatic adenocarcinomas, it may provide further evidence for the significance of dietary factors in the carcinogenesis of these two neoplasms in humans.

### Pancreatic Carcinoma and Colon Carcinoma

A total of 6 articles on synchronous pancreatic and colon carcinomas were retrieved (23, 31–35). Among these reports, Karak administered a combined treatment of adjuvant chemotherapy consisting of 5-FU and leucovorin following colectomy combined with pancreaticoduodenectomy. Follow-up showed that the patients had a relatively good prognosis.

The associations between pancreatic carcinoma and other carcinomas of the lung, kidney, cervix, ovary, prostate, thyroid, etc. are weak and have been less frequently reported. However, many researchers have found that MPCs involve concurrent alterations in multiple gene pathways where both hypermethylation of tumor suppressor genes and hypomethylation of tumor-promoting genes occur; the main treatment approach is combination therapy, including surgery, radiotherapy and chemotherapy (30, 36–47).

### Etiologies of MPCs

Some studies have speculated that the development of MPCs is associated with an unhealthy lifestyle, genetic susceptibility, side effects of chemotherapy and radiotherapy, weak immunity, etc. Moreover, as a tumor suppressor gene, inactivation of the TP53 gene plays a crucial role in the formation of tumors, and mutations in this gene exist in many solid tumors. Gerdes (11) reported that mutations of p16INK4a in the TP53 gene may be related to the occurrence of pancreatic cancer with multiple primary cancers. The patient in our case was overweight, with a BMI of 27.7, and high BMI is a well-known risk factor for the occurrence of malignant tumors, especially tumors of the digestive system (12). In addition, this patient had previously undergone partial thyroidectomy. Hormonal dysregulation may also be a risk factor for tumor development (12). Besides, another factor that could not be neglected is the weakened immunity associated with aging and increased tumor susceptibility (12).

### Therapeutic Options for MPCs

Most MPCs are found in the same pipeline system. In the diagnosis and treatment of multiple primary cancers, surgical exploration or biopsy should be used as soon as possible to make a clear diagnosis. When primary cancer or metastatic cancer cannot be distinguished, as long as the tumor is limited or isolated and the patient’s body condition allows, surgical resection should be performed to allow for the chance of cure. However, pancreatic cancer is an aggressive tumor with high mortality and poor prognosis. When pancreatic cancer patients have multiple primary cancers, priority should be given to a multidisciplinary combined treatment approach for pancreatic cancer. In this case, ESD was performed first to excise the esophagogastric junction lesions because this treatment was relatively easy to provide. Although we did not treat the tumor of the duodenal papilla or pancreatic tumor with surgery because pancreaticoduodenectomy could not be performed, intraoperative radiotherapy combined with postoperative adjuvant chemotherapy was used in a timely manner. Encouragingly, tumor progression or distant metastasis was not observed during the period of six cycles of chemotherapy, illustrating that intraoperative radiotherapy combined with postoperative adjuvant chemotherapy can effectively suppress advanced pancreatic cancer growth. Furthermore, this treatment result exemplifies the significant advantages of using multidisciplinary combined therapy for certain cancer patients.

The patient in our case received proton radiotherapy when she developed distant metastasis, and the level of CA199 presented a significant decrease (from 20000 U/ml to 700 U/ml). Proton radiotherapy is a type of external beam radiation therapy modality for the treatment of local disease that uses a proton beam to deliver a highly focused radiation dose to the tumor. In 1979, the University of Tsukuba began using proton cancer therapy with a booster accelerator for liver cancer, esophageal cancer, lung cancer, and brain tumors and achieved good curative effects (48). Proton radiotherapy has also gained popularity for the treatment of pancreatic cancer in recent years. Romaine, Michael and Dongha studied the efficacy of proton radiotherapy for the treatment of pancreatic cancer and found that patient survival was prolonged by this treatment (49–51). Here, our patient also exhibited a good treatment response. Although she ultimately passed away after an overall survival time of 18 months, this is much longer than the median survival time (6~11 months) of patients with locally advanced pancreatic cancer (52). The above evidence suggests that timely and effective multidisciplinary combined treatment for pancreatic cancer should be favored when pancreatic cancer patients are found to have multiple primary cancers. In a retrospective study of 147 cases of pancreatic resection for pancreatic cancer in Kumamoto University of Japan (53), it was found that when treating multiple primary cancers in patients with pancreatic cancer, priority should be given to pancreatic cancer treatment, and the postoperative outcomes and survival conditions were similar to those of pure pancreatic cancer patients. This also supports the validity of giving clinical priority to pancreatic cancer treatment.

In conclusion, the treatment of malignant tumors needs to adhere to the concept of multidisciplinary comprehensive treatment, and individualized precision therapy is especially valuable for patients with multiple primary cancers. Our experience and further accumulation of these cases will provide guidance and a reference for clinicians.

## Data Availability Statement

The original contributions presented in the study are included in the article/supplementary material. Further inquiries can be directed to the corresponding author.

## Ethics Statement

The studies involving human participants were reviewed and approved by Ethics Committee of the Cancer Hospital, Chinese Academy of Medical Sciences and Peking Union Medical College. The patients/participants provided their written informed consent to participate in this study.

## Author Contributions

YXD, YJD, and LZ wrote this paper. LZ and ZG collected relevant data of this patients. XZ and ZL followed up the patient. YXD, LZ, and CW managed the patient. CW and YXD revised this paper. All authors contributed to the article and approved the submitted version.

## Funding

This work was supported by the National Natural Science Foundation of China (81972314 and 81802463).

## Conflict of Interest

The authors declare that the research was conducted in the absence of any commercial or financial relationships that could be construed as a potential conflict of interest.

## Publisher’s Note

All claims expressed in this article are solely those of the authors and do not necessarily represent those of their affiliated organizations, or those of the publisher, the editors and the reviewers. Any product that may be evaluated in this article, or claim that may be made by its manufacturer, is not guaranteed or endorsed by the publisher.

## References

[1] Liu Z Liu C Guo W Li S Bai O. Clinical Analysis of 152 Cases of Multiple Primary Malignant Tumors in 15,398 Patients With Malignant Tumors. PloS One (2015) 10(5):e0125754. doi: 10.1371/journal.pone.0125754 25945938 PMC4422700

[2] Vogt A Schmid S Heinimann K Frick H Herrmann C Cerny T. Multiple Primary Tumours: Challenges and Approaches, A Review. ESMO Open (2017) 2(2):e000172. doi: 10.1136/esmoopen-2017-000172 28761745 PMC5519797

[3] Herrmann C Cerny T Savidan A Vounatsou P Konzelmann I Bouchardy C. Cancer Survivors in Switzerland: A Rapidly Growing Population to Care for. BMC Cancer (2013) 13:287. doi: 10.1186/1471-2407-13-287 23764068 PMC3685597

[4] Rosso S De Angelis R Ciccolallo L Carrani E Soerjomataram I Grande E. Multiple Tumours in Survival Estimates. Eur J Cancer (2009) 45(6):1080–94. doi: 10.1016/j.ejca.2008.11.030 19121933

[5] Utada M Ohno Y Hori M Soda M. Incidence of Multiple Primary Cancers and Interval Between First and Second Primary Cancers. Cancer Sci (2014) 105(7):890–6. doi: 10.1111/cas.12433 PMC4317925 24814518

[6] Yen HH Chen CN Yeh CC Lai IR. Adjuvant Tegafur-Uracil (UFT) or S-1 Monotherapy for Advanced Gastric Cancer: A Single Center Experience. World J Surg Oncol (2021) 19(1):124. doi: 10.1186/s12957-021-02233-2 33865416 PMC8053033

[7] Moertel CG Dockerty MB Baggenstoss AH. Multiple Primary Malignant Neoplasms. II. Tumors of Different Tissues or Organs. Cancer (1961) 14:231–7. doi: 10.1002/1097-0142(196103/04)14:2<231::aid-cncr2820140203>3.0.co;2-2 13771653

[8] Riall TS Stager VM Nealon WH Townsend CM Kuo YF Goodwin JS. Incidence of Additional Primary Cancers in Patients With Invasive Intraductal Papillary Mucinous Neoplasms and Sporadic Pancreatic Adenocarcinomas. J Am Coll Surgeons (2007) 204(5):803–14. doi: 10.1016/j.jamcollsurg.2007.01.015 17481488

[9] Hackert T Tjaden C Müller S Hinz U Hartwig W Strobel O. Extrapancreatic Malignancies in Patients With Pancreatic Cancer: Epidemiology and Clinical Consequences. Pancreas (2012) 41(2):212–7. doi: 10.1097/MPA.0b013e3182240602 21934549

[10] Lubezky N Ben-Haim M Lahat G Marmor S Solar I Brazowski E. Intraductal Papillary Mucinous Neoplasm of the Pancreas: Associated Cancers, Family History, Genetic Predisposition? Surgery (2012) 151(1):70–5. doi: 10.1016/j.surg.2011.06.036 21975290

[11] Nanashima A Kondo H Nakashima M Abo T Arai J Ishii M. Clinicopathological Characteristics of Multiple Primary Cancers in Hepatobiliary and Pancreas Malignancies. Anticancer Res (2015) 35(2):1073–83. 25667496

[12] Gerdes B Bartsch DK Ramaswamy A Kersting M Wild A Schuermann M. Multiple Primary Tumors as an Indicator for P16ink4a Germline Mutations in Pancreatic Cancer Patients? Pancreas (2000) 21(4):369–75. doi: 10.1097/00006676-200011000-00007 11075991

[13] Sasaki M. Case of Double Cancer With Reticuloendothelioma of the Liver and Carcinoma Arising From the Head of Pancreas. Nihon Rinsho. Japanese J Clin Med (1965) 23(10):2056–62. 5329398

[14] Zhang W Xiao F Li J Guo X Lin Z Huang Z. Rare Heterochronous Liver and Pancreatic Multiple Primary Cancers: A Case Report and Literature Review. Seltene Metachrone Multiple Primäre Tumore Der Leber Und Der Bauchspeicheldrüse: Ein Fallbericht Und Eine Literaturübersicht. Z fur Gastroenterol. (2020) 58(11):1094–8. doi: 10.1055/a-1160-6082 32380555

[15] Yoshii K Imaizumi T Miura O Nakasako T Hasegawa M Ogata S. A Case of Double Primary Carcinomas of the Pancreatic Head and Intrapancreatic Bile Duct, Hardly Diagnosed Preoperatively. Nihon Shokakibyo Gakkai zasshi = Japanese J gastro-enterol. (1989) 86(9):2260–4. 2685427

[16] Childs LC Harris MJ Lucas BA Kenady DE. Primary Biliary and Pancreatic Carcinoma After Renal Transplantation. South Med J (1990) 83(7):849–50. doi: 10.1097/00007611-199007000-00031 2164713

[17] Ueda N Nagakawa T Ohta T Kayahara M Ueno K Konishi I. Synchronous Cancer of the Biliary Tract and Pancreas Associated With Anomalous Arrangement of the Pancreaticobiliary Ductal System. J Clin Gastroenterol (1992) 15(2):136–41. doi: 10.1097/00004836-199209000-00011 1401825

[18] Nishihara K Tsuneyoshi M Shimura H Yasunami Y. Three Synchronous Carcinomas of the Papilla of Vater, Common Bile Duct and Pancreas. Pathol Int (1994) 44(4):325–32. doi: 10.1111/j.1440-1827.1994.tb03371.x 8044300

[19] Sato K Maekawa T Yabuki K Tamasaki Y Maekawa H Kudo K. A Case of Triple Synchronous Cancers Occurring in the Gallbladder, Common Bile Duct, and Pancreas. J Gastroenterol (2003) 38(1):97–100. doi: 10.1007/s005350300014 12560930

[20] Maurea S Corvino A Imbriaco M Avitabile G Mainenti P Camera L. Simultaneous non-Functioning Neuroendocrine Carcinoma of the Pancreas and Extra-Hepatic Cholangiocarcinoma. A Case of Early Diagnosis and Favorable Post-Surgical Outcome. JOP J pancreas (2011) 12(3):255–8. 21546703

[21] Bansal A Thung SN Zhu H Schwartz M Lewis S. Synchronous Pancreatic Adenocarcinoma and Intrahepatic Cholangiocarcinoma Arising in the Context of Intraductal Papillary Neoplasms. Clin Imaging (2016) 40(5):897–901. doi: 10.1016/j.clinimag.2015.12.019 27183137

[22] Vijayaraj P Chandrasekar S Kalayarasan R Pottakkat B. Double Trouble: Synchronous Adenocarcinoma of Gallbladder and Pancreas. J gastrointestinal Cancer (2018) 49(3):358–60. doi: 10.1007/s12029-017-9922-0 28168400

[23] Eriguchi N Aoyagi S Hara M Okuda K Tamae T Fukuda S. Synchronous or Metachronous Double Cancers of the Pancreas and Other Organs: Report on 12 Cases. Surg Today (2000) 30(8):718–21. doi: 10.1007/s005950070083 10955735

[24] M’sakni I Rammeh S Chelbi E Sayari S Zaouech A Baltagi-Ben Jilani S. Adénocarcinome Et Tumeur Stromale Digestifs: Association Fortuite Ou Mécanisme Oncogène Commun? A Propos De Deux Observations [Adenocarcinoma and Gastro-Intestinal Stromal Tumor: Fortuitous Association or a Single Carcinogenic Agent? A Report of 2 Cases]. Annales chirurgie (2006) 131(8):464–7. doi: 10.1016/j.anchir.2006.01.011 16527243

[25] Muroni M D’Angelo F Pezzatini M Sebastiani S Noto S Pilozzi E. Synchronous Gastric Adenocarcinoma and Pancreatic Ductal Adenocarcinoma. Hepatobiliary pancreatic Dis Int HBPD Int (2010) 9(1):97–9. 20133238

[26] Dasanu CA Mesologites T Trikudanathan G. Synchronous Tumors: Adenosquamous Carcinoma of Pancreas and GIST of Stomach. J gastrointestinal Cancer (2011) 42(3):186–9. doi: 10.1007/s12029-010-9187-3 20623381

[27] Kim JS Chung CY Park HC Myung DS Cho SB Lee WS. Synchronous Quadruple Primary Tumors of Thyroid, Breast, Pancreas, and Stomach: A Case Report. Anticancer Res (2013) 33(5):2135–8. 23645766

[28] Kourie HR Markoutsaki N Roussel H Rahmi G van der Stiegel M Palazzo L. Double Pancreatic and Gastric Adenocarcinomas: A Rare Association. Clinics Res Hepatol Gastroenterol (2013) 37(6):e137–40. doi: 10.1016/j.clinre.2012.09.008 23158953

[29] Ghothim M Havlík R Skalický P Klos D Vrba R Strážnická J. Synchronní Nádorové Duplicity Pankreatu a Žaludku/Ledviny a Jejich Léčba [Synchronous Cancer Duplicities of Pancreas and Stomach/Kidney and Their Surgical Treatment]. Rozhledy v chirurgii: mesicnik Ceskoslovenske chirurgicke spolecnosti (2015) 94(6):251–5. 26174345

[30] O’Brien ME Urbanski SJ. Coexisting Pancreatic and Breast Adenocarcinomas: Is There an Association? Pancreas (1986) 1(2):191–4. doi: 10.1097/00006676-198603000-00015 3033632

[31] Karak PK Mukhopadhyay S Singh SP Berry M. Multiple Primary Coexisting Malignancy in Young Patients. Trop gastroenterol.: Off J Digest. Dis Foundation (1994) 15(2):99–103. 7831723

[32] Joo YE Kim HS Choi SK Rew JS Kim SJ Park CS. Synchronous Pancreatic and Colon Primary Cancers. J Clin Gastroenterol (2001) 33(1):91–2. doi: 10.1097/00004836-200107000-00029 11418808

[33] Brinster DR Raper SE. Synchronous Colon and Pancreatic Cancers in a Patient With Peutz-Jeghers Syndrome: Report of a Case and Review of the Literature. Surgery (2004) 135(3):352–4. doi: 10.1016/s0039-6060(03)00379-9 14976486

[34] Li Destri G Giarrizzo A Bellavia N Milazzotto R Frattalone ME Scilletta B. Synchronous Double Cancers of the Colon and the Pancreas: A Case Report. Eur Rev Med Pharmacol Sci (2014) 18(2 Suppl):28–31. 25535188

[35] Couch LL Watlington JR Stanley JD Jean RJ. Two Primary Adenocarcinomas in a Single Patient: A Primary Colonic Adenocarcinoma and a Primary Pancreatic Adenocarcinoma Arising From Ectopic Pancreatic Tissue. Am surgeon (2019) 85(9):e439–40. doi: 10.1177/000313481908500903 31638530

[36] Langeron P Saout J Muanza-Nkashama E Béghin B. Cancer Double Simultané: Col Utérin Et Pancréas [Simultaneous Double Cancer: Cervix of Uterus and Pancreas]. J Des Sci medicales Lille (1972) 90(2):59–66. 5050411

[37] Sasaki E Kushida S Okinaka T Sasaki K Abe S. Simultaneous Double Cancer: Cervix of Uterus and Pancreas. Gan no rinsho. Japan J Cancer Clinics (1969) 15(2):203–6. 5814514

[38] Kawaura Y Ohike E Hirano M Iwa T Haratake J. An Autopsy Case of Pancreatic Cancer With Carcinoid Tumor of the Appendix. Gan No Rinsho. Japan J Cancer Clinics (1983) 29(8):939–42. 6887529

[39] Hori N Kinoshita N Hoshina A Kato M Nishii M Tajima K. Two Cases of Triple Primary Neoplasm and Two Cases of Quadruple Primary Neoplasm Including Bladder Cancer. Hinyokika Kiyo. Acta urologica Japonica (1985) 31(10):1807–11. 4091130

[40] Niv Y Abu-Avid S Oren M. Adenocarcinoma of Pancreas and Duodenum Associated With Cutaneous Neurofibromatosis. Am J Med (1987) 82(2):384–5. doi: 10.1016/0002-9343(87)90096-9 3101498

[41] Niwa K Yoshimi N Sugie S Sakamoto H Tanaka T Kato K. A Case of Double Cancer (Pancreatic and Ovarian Adenocarcinomas) Diagnosed by Exfoliative and Fine Needle Aspiration Cytology. Japanese J Clin Oncol (1988) 18(2):167–73. 3379784

[42] Montag AG Fossati N Michelassi F. Pancreatic Microcystic Adenoma Coexistent With Pancreatic Ductal Carcinoma. A Report of Two Cases. Am J Surg Pathol (1990) 14(4):352–5. doi: 10.1097/00000478-199004000-00006 2157344

[43] Aurello P Milione M Dente M D’Angelo F Nigri G Del Gaudio M. Synchronous Carcinosarcoma of the Intrapancreatic Bile Duct and Carcinoma *in Situ* of Wirsung Duct: A Case Report. Pancreas (2008) 36(1):95–7. doi: 10.1097/MPA.0b013e31813e64b5 18192890

[44] Ozsoy O Fioretta G Ares C Miralbell R. Incidental Detection of Synchronous Primary Tumours During Staging Workup for Prostate Cancer. Swiss Med weekly (2010) 140(15-16):233–6. 10.4414/smw.2010.12976 20407958

[45] Power DG Shia J Allen PJ Jarnagin WR O’Reilly EM. Synchronous Epithelial and Neuroendocrine Cancers of the Pancreas: Case Series of a Rare Occurrence. Clin colorectal Cancer (2011) 10(2):146–50. doi: 10.1016/j.clcc.2011.03.013 21859569

[46] Olgyai G Haulik L Oláh A. Primer Pancreas- És Vesetumor Szinkron Reszekciója–Esettanulmány [Double Resection for Synchronous Pancreatic and Renal Cell Cancer–Case Report]. Magyar sebeszet (2004) 57(5):287–9. 15907012

[47] Araki K Shimura T Kobayashi T Saito K Wada W Sasaki S. Mixed Ductal-Endocrine Carcinoma of the Pancreas Occurring as a Double Cancer: Report of a Case. Int Surg (2011) 96(2):153–8. doi: 10.9738/cc8.1 22026308

[48] Dinesh Mayani D. Proton Therapy for Cancer Treatment. J Oncol Pharm Pract (2011) 17(3):186–90. doi: 10.1177/1078155210375858 20634263

[49] Nichols RC Huh S Li Z Rutenberg M. Proton Therapy for Pancreatic Cancer. World J gastrointestinal Oncol (2015) 7(9):141–7. doi: 10.4251/wjgo.v7.i9.141 PMC4569591 26380057

[50] Rutenberg MS Nichols RC. Proton Beam Radiotherapy for Pancreas Cancer. J gastrointestinal Oncol (2020) 11(1):166–75. doi: 10.21037/jgo.2019.03.02 PMC7052755 32175120

[51] Lee D Komatsu S Terashima K Toyama H Matsuo Y Takahashi D. Surgical Spacer Placement for Proton Radiotherapy in Locally Advanced Pancreatic Body and Tail Cancers: Initial Clinical Results. Radiat Oncol (2021) 16(1):3. doi: 10.1186/s13014-020-01731-z 33407648 PMC7788736

[52] Cardenes HR Chiorean EG Dewitt J Schmidt M Loehrer P. Locally Advanced Pancreatic Cancer: Current Therapeutic Approach. oncol. (2006) 11(6):612–23. doi: 10.1634/theoncologist.11-6-612 16794240

[53] Yamamura K Hashimoto D Kitano Y Kuroda D Eto T Arima K. Multiple Primary Cancers in Patients With Pancreatic Cancer. Am surgeon (2018) 84(12):e514–6. doi: 10.1177/000313481808401205 30606360

